# Elevated expression of RNA methyltransferase BCDIN3D predicts poor prognosis in breast cancer

**DOI:** 10.18632/oncotarget.9656

**Published:** 2016-05-28

**Authors:** Ling Yao, Yayun Chi, Xin Hu, Shan Li, Feng Qiao, Jong Wu, Zhi-Ming Shao

**Affiliations:** ^1^ Department of Breast Surgery, Fudan University, Shanghai Cancer Center and Cancer Institute, Shanghai, P.R. China; ^2^ Department of Oncology, Shanghai Medical College, Fudan University, Shanghai, P.R. China; ^3^ Institutes of Biomedical Science, Fudan University, Shanghai, P.R. China

**Keywords:** BCDIN3D, breast cancer, TNBC

## Abstract

**Background:**

BCDIN3D is a member of the Bin3 methyl-transferase family that targets the 5' mono-phosphate of nucleic acids. Although BCDIN3D has been shown to increasetumorigenic phenotypes and invasiveness in MDA-MB-231 cells, its the clinical implications in breast cancer remain unclear.

**Methods:**

We screened for BCDIN3D using tissue microarrays constructed from 250 patients who were histologically confirmed to have invasive ductal breast carcinoma at the Fudan University Shanghai Cancer Center.

**Results:**

The survival analysis by Kaplan-Meier and Cox regression showed that BCDIN3D expression level served as a prognostic factor for disease-free survival (*P* = 0.042). The prognostic value of BCDIN3D was most significant in triple-negative breast cancer (TNBC) patients (*P* = 0.007).

**Conclusions:**

BCDIN3D might serve as an important prognostic factor for TNBC patients.

## BACKGROUND

Breast cancer is the most common cancerin women, and accountfor approximately 23% of allcancer cases and approximately 14% of cancer deaths[1]. It is a heterogeneous disease embracing several different phenotypes [2], including luminal A, luminal B, human epidermal growth factor receptor 2 (HER2)-enriched, and triple-negative breast cancer (TNBC) [3]. Breast cancer results from the accumulation of genetic and epigenetic alterations[4, 5]. Epigenetic alterations, as defined by modifications of DNA, histones and coding/noncoding RNAs [6, 7], also contribute to various phases of neoplastic development including initiation, promotion, invasion, metastases.

RNA modification is a kind of epigenetic regulation of gene expression, analogous to DNA methylation and histone modification. [8]. Methylation is a ubiquitous modification that affects several residues/sites in molecules [9]. Methyl-transfer reactions to RNA nucleotides are catalyzed by a variety of RNA-MTases that include more than 60 members with hundreds of homologs, and which have so far been divided into four super-families[10]. BCDIN3D, a member of the Bin3 methyltransferase family, share homology within their putative S-adenosyl Methionine (SAM) binding motif from S. pombe to human. SAM (S-adenosylmethionine) is well known as the methyl donor for methyl-transferases that modify DNA, RNA, histones[11].

BCDIN3D is a methyltransferase that targets the 5'mono-phosphate of nucleic acids. As depletion of BCDIN3D in the MDA-MB-231 cells abolishes anchorage-independent growth and decreases invasiveness in MDA-MB-231 cells, but not growth ormigration [12], its prognostic value may be of interestHerein, we investigated BCDIN3D expression and its association with tumor progression and clinical outcome in a cohort of 250 patients who had undergone surgery for breast cancer in eastern Chinese women population.

## RESULTS

### Patient characteristics

Clinical-pathological characteristics of the study cohort are summarized in Table 1. Patients' median age at diagnosis was 52.02 years. After a mean follow-up time of 83.36 months, 55 of the 250 patients showed recurrence of disease. In univariate analysis, histological grade (HR, 1.761; 95% CI, 1.041-2.980; *P* = 0.035), tumor size (HR, 1.715; 95% CI, 1.086-2.708; *P* = 0.021), metastatic nodes (HR, 2.231;95% CI, 1.304-3.819; *P* = 0.003), were significantly associated with higher risk of recurrence and reached statistical significance as expected, however, only the association with tumor size and metastatic nodes remained statistically significant inmultivariate analysis (Table 3).

**Table 1:** Clinical-pathological characteristics of the study cohort

Clinical-pathological characteristics	No.	Percentage (%)
Age (years) (mean 52.02, SD 9.642, median 51, range 29-85)		
≤50	120	48.0
>50	130	52.0
Menopausal status		
Pre	108	43.2
Post	142	56.8
TNM Stage		
I	74	29.6
II	132	52.8
III	42	16.8
Unknown	2	0.8
Histological grade		
I	5	2.0
II	184	73.6
III	61	24.4
Tumor size		
T1 (≤2cm)	115	46.0
T2 (>2-5cm)	120	48.0
T3 (>5cm)	13	5.2
Unknown	2	0.8
Node status		
Negative	151	60.4
Positive	97	38.8
Unknown	2	0.8
ER status		
Negative	143	57.2
Positive	106	42.4
Unknown	1	0.4
PR status		
Negative	184	73.6
Positive	63	25.2
Unknown	3	1.2
HER-2 status		
Negative	148	59.2
Positive	101	40.4
Unknown	1	0.4
Molecular subtype^a^		
Luminal like	106	42.4
HER-2 Positive	42	16.8
Triple Negative	100	40.0
Unknown	2	0.8

Abbreviations: ER, estrogen receptor; PR, progesterone receptor; HER-2, human epidermal growth factor receptor 2; SD, standard deviation;

a. Definition of breast cancer molecular subtypes: luminal like (ER and/or PR positive, any HER-2 status), HER-2 Positive (ER and PR negative, HER-2 positive) and triple negative (ER negative, PR negative and HER-2 negative)

**Table 3:** Univariate and multivariate analysis of factors related to disease-free survival (DFS) in breast cancer patients

Variables	DFS			
	Univariate Analysis		Multivariate Analysis	
	HR(95% CIs)	*P*	HR(95% CIs)	*P*
Age	0.815(0.552-1.534)	0.939	0.971(0.554-1.700)	0.917
Histological grade	1.761(1.041-2.980)	**0.035**	1.749(0.965-3.170)	0.065
Tumor Size	1.715(1.086-2.708)	**0.021**	1.648(1.042-2.606)	**0.033**
Metastatic nodes	2.231(1.304-3.819)	**0.003**	2.158(1.236-3.769)	**0.007**
ER	0.786(0.456-1.356)	0.388	1.491(0.713-3.117)	0.288
PR	0.408(0.185-0.903)	**0.027**	0.372(0.144-0.960)	**0.041**
HER-2	0.936(0.548-1.600)	0.809	0.754(0.412-1.378)	0.358
BCDIN3D	1.754(1.012-3.039)	**0.045**	1.904(1.081-3.354)	**0.026**

Abbreviations: HR, hazard ratio; CIs, confidence intervals; ER, estrogen receptor; PR, progesterone receptor; HER-2, human epidermal growth factor receptor 2;

### Expression patterns of BCDIN3D in breast cancer patients

**Figure 1:**
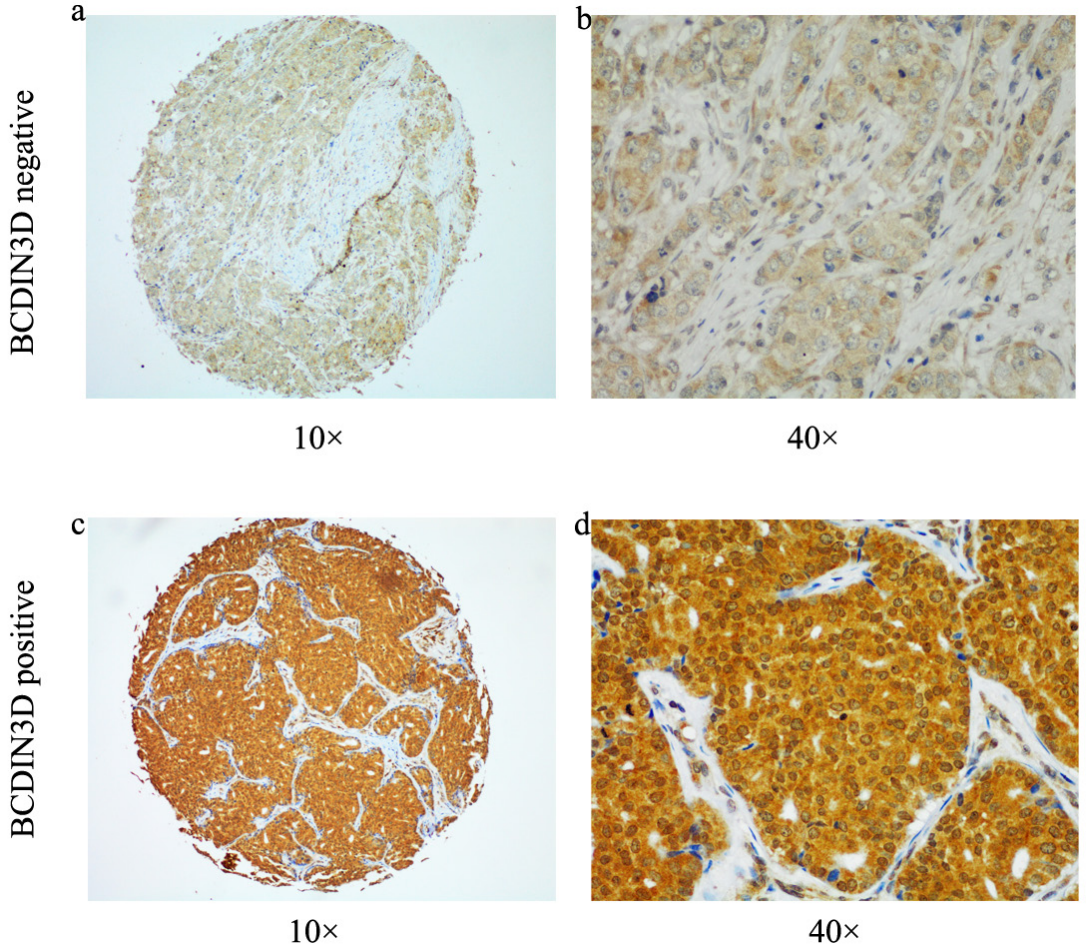
Representative image of immunohistochemical BCDIN3D staining were shown in both small pictures (×100 magnification) and large (×400 magnification) **a.**-**b.** Negative for BCDIN3D. **c.**-**d.** Positive for BCDIN3D.

In this cohort of 250 patients, TMAswere immunostained for BCDIN3D (representative images, Figure 1). The specificity of the antibody against BCDIN3D was confirmed by Western blot of human cell lysis. Positive staining of BCDIN3D was detected in 49.6% of tumors according to the scoring criterion described above (*n* = 124; 49.6% positive, 50.4% negative; Figure 1a-1d). We further analyzed relationships between clinicopathologic features and the expression level of BCDIN3D. The percentages of positive staining of protein were consistent across all subsets of patients (Table 2).

**Table 2:** Correlations between patients' characteristics and expression status of BCDIN3D

Clinical-pathological characteristics		Cases	BCDIN3D		
			-	+	*P*^a^
		250	126	124	
Percentage (%)			50.40%	49.60%	
Age(years)					
≤50		120	62	58	0.521
>50		130	64	66	
Menopausal status					
Pre		108	54	54	1.000
Post		142	72	70	
TNM Stage					
I		74	32	42	0.250
II		132	73	59	
III		42	21	21	
Histological grade					
I		5	1	4	0.334
II		184	92	92	
III		61	33	28	
Tumor size					
T1 (≤2cm)		115	52	63	0.318
T2 (>2-5cm)		120	66	54	
T3 (>5cm)		13	8	5	
Node status					
Negative		151	75	76	0.697
Positive		97	51	46	
ER status					
Negative		143	69	74	0.522
Positive		106	56	50	
PR status					
Negative		184	88	96	0.243
Positive		63	36	27	
HER-2 status					
Negative		148	78	70	0.368
Positive		101	47	54	

Abbreviations:ER, estrogen receptor; PR, progesterone receptor; HER-2, human epidermal growth factor receptor 2;

a. Pearson χ2 test

### BCDIN3D was identified as a significant prognostic factor in breast cancer patients, especially in triple-negative breast cancers (TNBC)

**Figure 2:**
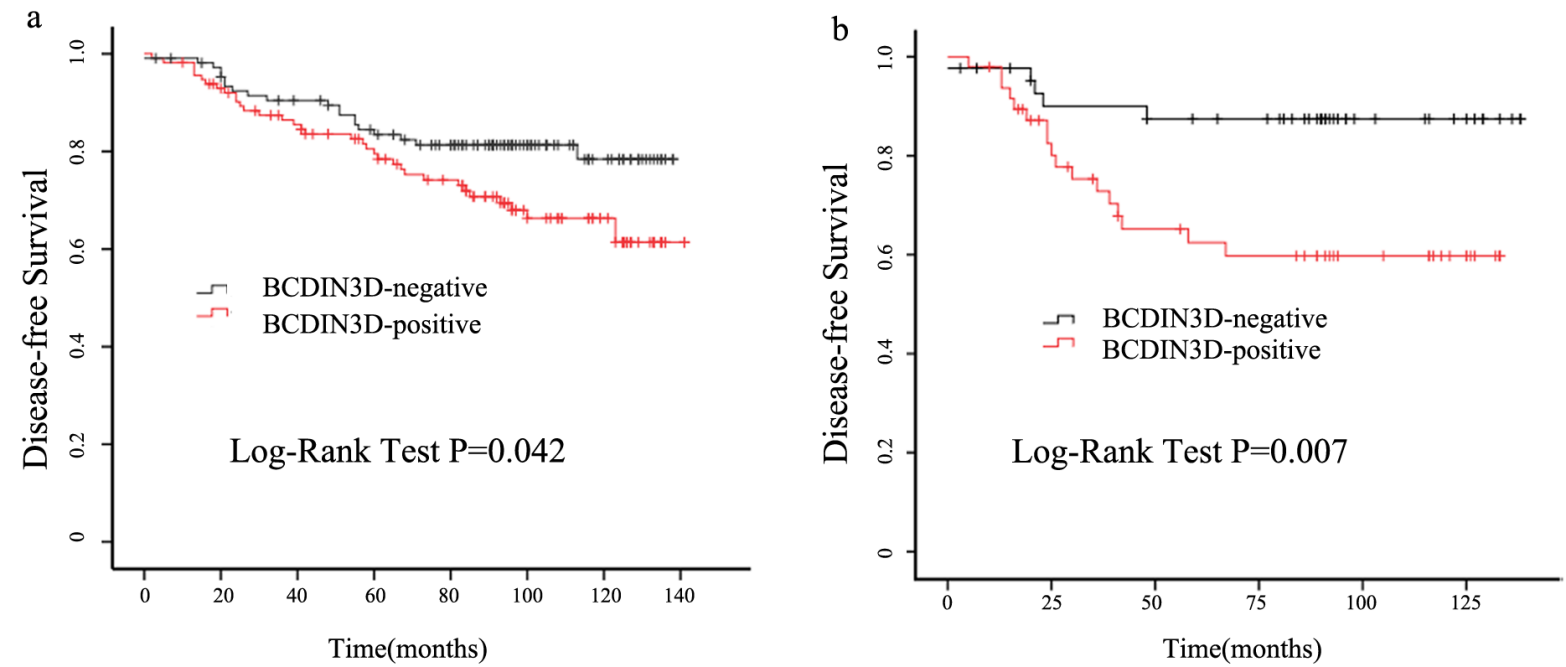
Elevated expression of BCDIN3D predictsworse clinical outcome in breast cancer patients, especially in TNBC **a.** Cumulative disease-free survival curves of patients with positive or negative expression of BCDIN3D in breast cancer patients. **b.** Cumulative disease-free survival curves of patients with positive or negative expression of BCDIN3D in TNBC patients.

Both univariate and adjusted multivariate survival analyses showed significant differences in DFS between the BCDIN3D positive and negative groups. BCDIN3D positive group having a significantly higher incidence of disease eventsin both univariate analysis (HR = 1.754; 95% CI: 1.012-3.039; *P* = 0.045) and multivariate analysis. (HR = 1.904; 95% CI: 1.081-3.354; *P* = 0.026) (Table 3). The BCDIN3D positive group also showed worse DFS in the Kaplan-Meier survival analysis *(P* = 0.042; Figure 2a). Thus, these results indicate that BCDIN3D expression isdirect associated with breast cancer recurrence. Then we analyzed the relationship between BCDIN3D expression and survival according to the different breast cancer subtypes. We found the prognostic value of BCDIN3D for DFS was most significant among patients with TNBC *(P* = 0.007; Figure 2b). In the TNBCsubset, patients with positive BCDIN3D staining showed a higher likelihood of occurence of disease events (HR = 3.584; 95% CI: 1.319-9.737; *P* = 0.012) in univariate analysis and remain the same trend in multivariate analysis. (HR = 3.719; 95% CI: 1.345-10.283; *P* = 0.011)(Table 4).

**Table 4:** Univariate and multivariate analysis of factors related to disease-free survival (DFS) in TNBC patients

Variables	DFS			
	Univariate Analysis		Multivariate Analysis	
	HR(95% CIs)	*P*	HR(95% CIs)	*P*
Age	0.967(0.417-2.238)	0.937	1.192(0.503-2.824)	0.690
Histological grade	1.273(0.553-2.927)	0.570	0.953(0.392-2.315)	0.915
Tumor Size	2.837(1.381-5.825)	**0.005**	1.950(1.023-3.719)	**0.042**
Metastatic nodes	3.195(1.364-7.486)	**0.007**	3.157(1.265-7.880)	**0.014**
BCDIN3D	3.584(1.319-9.737)	**0.012**	3.719(1.345-10.283)	**0.011**

Abbreviations: HR, hazard ratio; CIs, confidence intervals; ER, estrogen receptor; PR, progesterone receptor; HER-2, human epidermal growth factor receptor 2;

## DISCUSSION

Breast Cancer, is now known to involve epigenetic abnormalities along with genetic alterations [13]. Epigenetic modifications are early events in breast carcinogenesis and could be usefulfor early detection, prognosis, and targeted therapy of breast cancer [14]. Post-transcriptional gene regulation by noncoding RNA commonly referred as microRNAs is a kind of epigenetic regulation [15, 16]. It has become one of the most dynamic and fast-growing fields in science. Despite the rapid advance of RNA research, the enzymes that post-transcriptionally modify RNA have been less investigated[17].

A post-transcriptional modification enzyme BCDIN3D O-methylates the 5′ terminal mono-phosphate group of the precursor of some miRNA, which increasedtumorigenic phenotypes and cells invasiveness[12]. To our knowledge, no one else has explored its clinical significance. Our study is the first to evaluate the prognostic value of BCDIN3D in breast cancer patients. Our finding that patients with BCDIN3D positive tumors had worse DFS than the BCDIN3D negative group supports previous biological function study results from other researchers. Interestingly, this association was found to be most significant among patients with TNBC. TNBC breast tumors lack ER, PR, and HER-2 expression and occupy 15-20% of all breast cancers. It is generally more aggressive, has higher rates of relapse and decreased overall survival [18]. As few biomarkers are widely considered to be predictive for TNBC prognosis, and thus predictive factors for TNBC are urgently need. Our findings suggest that BCDIN3D might serve as an important prognostic factor for TNBC patients.

Our study had several limitations, including the small data set, few cases of stage I patients, lack of validation in an independent series of cases, and the composition of the study cohort which did not exactly represent that of the breast cancer population. Howeverour study was based on the use of TMAs, which can guarantee the consistency and coherence of these factors.

## CONCLUSIONS

In conclusion, our results associate, for the first time, higher BCDIN3D levels with worse DFS, especially in triple-negative breast cancer population, which suggests its potential use as a predictive biomarker. Furthermore, as more future basic and clinical studies uncover the underlying mechanism in this pattern, BCDIN3D could emerge as a desperately needed therapeutic targetin triple-negative breast cancer.

## MATERIALS AND METHODS

### Patients and specimens

250 primary breast cancer samples of stage I to III invasive ductal carcinoma cases were collected randomly at the Department of Breast Surgery in Fudan University Shanghai Cancer Center (FDUSCC, Shanghai, P.R. China) between August 2001 and March 2006. Their clinical-pathological characteristics and the systemic therapies are presented in Table 1. In this retrospective cohort study, they have been followed regularly, and 227 cases obtained the clinical outcome, with the last update in September 2013. Their median follow-up time was 96 months.

### Ethics statement

This study was approved by the Ethics Committee of FDUSCC, and each participant signed an informed consent document.

### Tissue microarrays (TMAs)

TMAs were constructed from formalin-fixed, paraffin-embedded samples of carcinomas obtained from the250 breast cancer patients. A hematoxylin- and eosin-stained section of each tumor block was used to mark representative tumor regions. Tissue cylinders with a diameter of 2 mm were punched from the above regions and transferred to recipient array blocks using a Tissue Micro Arrayer (Beecher Instruments, Sliver Springs, MD, USA). TMAs werecomposed of duplicate cores from different areas of the same tumor to compare staining patterns.

### IHC experimental procedures

The tissue micro arrays were subjected to immunohistochemical staining for BCDIN3D, using a 2-step protocol (GTVisionTMIII). The primary antibodies used were monoclonal BCDIN3D antibody (Santa Cruz Biotechnology, CA, USA sc-390348). The specificity of the BCDIN3D antibody was validated by western blot (Figure S1). The TMAs were deparaffinized with xylene, and rehydrated with an ethanol gradientThe sections were then rinsed with phosphate buffer solution (PBS) for immunohistochemical staining. For antigen retrieval the sections were immersed in 0.01 M tris sodium citrate pH 6.0 and boiled at 121 °C for 5 min followed by 2 min simmering. All slides were incubated with nonspecific staining blocking agent for 20 minutes to quench endogenous peroxidase activity and then with anti-BCDIN3D (1:200) at 4 °C overnight. Primary antibodies were detected by HRP-conjugated secondary antibodies followed by colorimetric detection with 3, 3-diaminobenzidine (DAB). The TMAs were then counterstained with Gill hematoxylin and dehydrated in an ascending ethanol series before clearing with xylene and mounting under a coverslip.

### Evaluation of immunohistochemical variables

TMAs were stained and scored semi-quantitatively. The score used in all subsequent analysis was the average across the available cores. Staining was graded for intensity of staining (0, no staining; 1+, faint/equivocal; 2+, moderate; 3+, strong) and percentage of cells stained (0, no staining; 1+, < 10% of cells stained; 2+, 10% -50% of cells; and 3+, >50% of cells stained). For this study, SI ≥3 was defined as positive staining, and SI ≤2 was defined as negative staining. Scoring was reviewed in parallel by two experienced breast disease pathologistswho were blinded to all clinical data.

### Statistical analyses

Disease-free survival(DFS) was defined as the time between the date of the primary surgery to the date of relapse/breast cancer specific death or September 2013. The first recurrence of disease at a local, regional, or distant site; diagnosis of contralateral breast cancer; and the breast cancer specific death were considered DFS events. Patients with study end date and loss of follow-up were considered to be censored. Correlations between clinical-pathological parameters and BCDIN3D were tested using the Chi-squared test. Survival outcomes were estimated using the Kaplan-Meier method and were compared between the groups using log-rank statistics. Univariate and multivariate analyseswere carried out using the Cox risk proportion model. Statistics was analyzed using SPSS (version 13.0; SPSS Company). All P values are two-sided; P value less than 0.05 was considered significant. All analyses were based on the observed data with the assumption that missing data were completely at random.

## SUPPLEMENTARY MATERIAL FIGURE


