# Supplementary material for: Elevated expression of RNA methyltransferase BCDIN3D predicts poor prognosis in breast cancer
Source: Oncotarget. 2016 May 28;7(33):53895–902. doi: 10.18632/oncotarget.9656 (PMC5288229; doi:10.18632/oncotarget.9656)
Supplement: Supplementary file 1 [file oncotarget-07-53895-s001.pdf]

Elevated expression of RNA methyltransferase BCDIN3D predicts poor prognosis in breast cancer

Supplementary Material

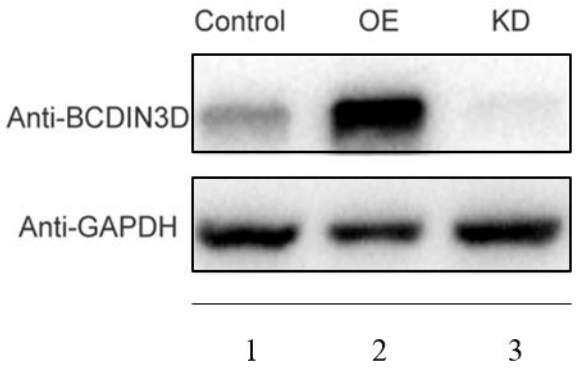

Fig S1. Western blot analyses of MDA-MB-231 cells, BCDIN3D OE MDA-MB-231 cells, and BCDIN3D KD MDA-MB-231 cells using anti-BCDIN3D and anti-GAPDH.

Lane 1: MDA-MB-231 cells;

lane 2: BCDIN3D OE MDA-MB-231 cells;

and lane 3: BCDIN3D KD MDA-MB-231 cells.
